# Comparison of cold and hot snaring polypectomy for small colorectal polyps: study protocol for a randomized controlled trial

**DOI:** 10.1186/s13063-018-2743-z

**Published:** 2018-07-06

**Authors:** Yen-Nien Chen, Li-Chun Chang, Chi-Yang Chang, Peng-Jen Chen, Chi-Yi Chen, Cheng-Hao Tseng, Han-Mo Chiu

**Affiliations:** 1 Department of Internal Medicine, National Taiwan University Hospital, Hsin-Chu Branch, Hsin-Chu, Taiwan; 2 Department of Internal Medicine, National Taiwan University Hospital, Taipei, Taiwan; 3 Health Management Center, National Taiwan University Hospital, Taipei, Taiwan; 4 Department of Internal Medicine, Fu Jen Catholic University Hospital, New Taipei City, Taiwan; 5 Division of Gastroenterology, Tri-Service General Hospital, National Defense Medical Center, Taipei, Taiwan; 6 Division of Gastroenterology and Hepatology, Department of Internal Medicine, Chia-Yi Christian Hospital, Chia-Yi, Taiwan; 7 Department of Gastroenterology and Hepatology, E-Da Hospital, Kaohsiung, Taiwan

**Keywords:** Cold snaring polypectomy, Small colorectal polyp, Delayed bleeding

## Abstract

**Background:**

Colorectal cancer remains a considerable challenge in healthcare nowadays. Most patients’ disease develops via the adenoma–carcinoma sequence; colonoscopy with polypectomy effectively reduces both mortality and incidence by removing precancerous adenomas. Previous studies showed that polypectomy without electrocautery (cold snaring polypectomy) is a safe and time-saving procedure to manage polyps < 10 mm. However, randomized controlled trials have failed to prove the superiority of cold snaring polypectomy for reducing the risk of delayed bleeding in comparison with hot snaring polypectomy, generally because of their low statistical power that was limited by sample sizes. In this study, we aim to compare the risk of delayed bleeding following cold and hot snaring polypectomy based on a large sample size.

**Methods:**

This is a prospective multicentre randomized controlled trial to compare cold and hot snaring polypectomy for the treatment of small colorectal polyps. A total of 4258 patients with small polyps (4–10 mm) will be randomized 1:1 to each group. Colonoscopy and polypectomy will be performed by 17 experienced endoscopists at six study sites. The randomization will be performed via an online website. Pathological examination using image-enhanced endoscopy with either narrow-band imaging or chromoendoscopy will be conducted to confirm optically and histologically that complete resections have been achieved, respectively. The primary outcome measurement is the risk of delayed bleeding. The secondary outcome measurements include the number of hemoclip applications, complete eradication confirmed optically and histologically, tissue retrieval rate, procedure time, emergency unit visits, and any adverse events such as immediate bleeding or perforation.

**Discussion:**

We hypothesize that cold snaring polypectomy can reduce the risk of delayed bleeding by avoiding thermal injury. In addition, this study will also compare cold and hot snaring polypectomy in terms of the complete eradication rate and procedure time. Based on data collected, we will demonstrate that cold snaring polypectomy is a safe, effective, and economic procedure for small colorectal polyps. The results will also provide additional data on which to develop recommendations for treating small colorectal polyps.

**Trial registration:**

ClinicalTrials.gov, NCT03373136. Registered on 29 November 2017.

**Electronic supplementary material:**

The online version of this article (10.1186/s13063-018-2743-z) contains supplementary material, which is available to authorized users.

## Background

Colorectal cancer (CRC) is the third most common cancer and is the fourth leading cause of cancer-related deaths worldwide [1]. The incidence of CRC has gradually increased worldwide, except in the United States [2], where the incidence rates have declined by about 3% per year among adults aged ≥ 50 years [3]. This trend primarily reflects the effects of wider CRC screening and removal of precancerous adenomas [4]. Moreover, the decreasing mortality associated with CRC has also been observed in many countries worldwide and probably can be attributed to the expansion of CRC screening, patients’ lifestyle modifications to reduce their risk factors, and advances in treatments [4, 5].

Colonoscopy is an important modality for screening CRC and polypectomy has been proven to significantly reduce the risks of CRC incidence and associated mortality [6–8]. Polypectomy is a safe procedure, but polypectomy-related complications such as bleeding and perforation do exist [9, 10]. Nearly 80% of screening-detected polyps are < 10 mm; therefore, an important issue regards how to manage these small polyps effectively, economically, and safely. Physicians have several ways of removing small polyps, including forceps biopsy and snare polypectomy. Both forceps biopsy and snare polypectomy can be further divided into hot and cold procedures that use electrocautery or manual manipulations, respectively [11]. Snare polypectomy without electrocautery, cold snaring polypectomy, was first described more than two decades ago and has been reported to be safe and effective [12–15]. Furthermore, previous studies also disclosed that cold snaring polypectomy can save more procedural time than hot snaring polypectomy [16–19].

Cold snaring polypectomy has been considered useful for reducing the risk of delayed bleeding because it avoids electrocautery-associated thermal injury. Nevertheless, previous studies have failed to demonstrate the superiority of cold snaring polypectomy compared with hot snaring polypectomy in terms of the risk of delayed bleeding. One meta-analysis included five randomized controlled trials (RCTs) that included 668 participants and disclosed that cold snaring polypectomy had a lower bleeding rate than hot snaring polypectomy, but the difference did not reach statistical significance [20]. Horiuchi et al. demonstrated that cold snaring polypectomy was associated with a significantly lower risk of immediate and delayed bleeding compared with hot snaring among patients who were taking an anticoagulant [15]. Because of their limited sample sizes and based on the lower risk of bleeding among high-risk individuals, the previous RCTs failed to demonstrate a reduced risk of bleeding in the group that underwent cold snaring polypectomy.

In this context, an RCT with a larger sample size is warranted to compare the risk of delayed bleeding between patients treated with cold versus hot snaring polypectomy techniques.

## Methods and design

### Study design

This trial is a prospective RCT comparing the risk of delayed bleeding following cold and hot snaring polypectomy for the treatment of small and diminutive colorectal polyps. This is a multicenter trial that will be conducted at National Taiwan University Hospital, Tri-Service General Hospital, Fu-Jen Catholic University Hospital, National Taiwan University Hospital, Hsin-Chu Branch, Chia-Yi Christian Hospital, and E-Da Hospital, all in Taiwan. These sites are located in the northern, central, and southern areas of Taiwan; it can provide adequate generalizability to represent the current clinical practice environment in Taiwan. The study was approved by the Institutional Review Board of the National Taiwan University Hospital (No. 201707022RINB) and it has also been registered at Clinical Trials.gov (NCT03373136). This trial protocol was written in accordance with the Standard Protocol Items: Recommendations for Interventional Trials (SPIRIT). The SPIRIT checklist has been included as Additional file 1.

### Study patients

**Fig. 1:**
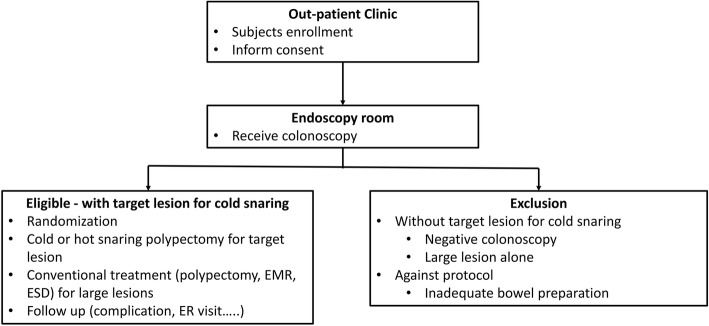
Study workflow. Patients will be enrolled and will sign the inform consent in the outpatient department. The colonoscopy will be performed in the endoscopy unit. Patients who have colorectal polyps sized 4–10 mm will be randomized into either cold or hot snaring polypectomy regardless of whether large colorectal polyps are present. Small-sized polyps will be removed by hot or cold snaring polypectomy, depending on the the patient’s allocation to the cold or hot snaring group, and large-sized polyps (> 10 mm) will receive hot snaring polypectomy, EMR, or ESD according to the colonoscopist’s decision

Patients scheduled for screening or surveillance colonoscopy will be prospectively screened for eligibility and 4258 patients will be enrolled during the study period. The workflow is shown in Fig. 1. The inclusion criteria are as follows: patients aged > 20 years; have an indication for colonoscopy; and have at least one colorectal polyp of 4–10 mm in diameter. Patients who fulfill at least one of the following exclusion criteria will be considered ineligible for this study: (1) age < 20 years; (2) any contraindication for colonoscopy or polypectomy; (3) pregnancy; or (4) inadequate bowel preparation that could interfere with the procedure or polypectomy. The patients will be enrolled into this trial and provide informed consent at outpatient clinic visiting; they will have enough time to consider whether to participate in this study. The patients’ enrollment will not be the same day of the colonoscopy procedure. In addition, the management of antithrombotic agents for enrolled patients will follow the updated American Society for Gastrointestinal Endoscopy (ASGE) guideline [21]. Patients who fulfill the inclusion criterion will be randomized into either cold or hot snaring polypectomy treatment groups.

### Endoscopists and equipment

Each procedure will be performed at the six study sites by one of the 17 endoscopists; each one collectively has experience with > 5000 colonoscopies.

Colonoscopes with a variable-stiffness function (CF-260 or 290 series; Olympus Medical Systems, Tokyo, Japan) will be used for all procedures. The following models of snare will be used for both cold and hot snaring polypectomy: Captivator-Small Hex 13 mm; Captivator II-Round 10 mm; and Captivator II-Round 15 mm (Boston Scientific, Boston, MA, USA). One of these snares will be applied for polypectomy of small polyps, depending on the size and morphology of the polyp and the preference of the endoscopists. Concurrent lesions > 10 mm will also be resected using snares other than the models just mentioned, depending also on the lesions’ size and morphology.

### Randomization

Randomization will be based on the appearance of the target lesions, polyps sized 4–10 mm, present at colonoscopy. Eligible patients will be centrally randomized 1:1 into either cold or hot snaring polypectomy by online software. The online software will be a web-based computer program accessed via an application on a smartphone (Interrand Inc., Ottawa, ON, Canada). Randomization procedures will be conducted by research assistants in the endoscopy units. The patients will be blinded about which treatment group they are randomized into during and after the procedures.

### Procedures

For both groups, the procedures for bowel preparation and colonoscopy insertion will take place as described in a previous study [22]. Colorectal polyps sized < 4 mm will be removed by forceps biopsy. Lesions sized 4–10 mm will be removed either by hot or cold snaring polypectomy based on the allocation arm. No matter hot or cold snaring polypectomy, saline or adrenaline solution injection to the lesion will not be performed before polypectomy. Lesions sized ≥ 10 mm will be removed by hot snaring polypectomy, endoscopic mucosal resection (EMR), or endoscopic submucosal dissection (ESD) as indicated and based on the feasibility, risk, and patients’ consent. Patients who are allocated to the cold snaring group will receive snaring polypectomy without electrocautery for all target lesions except for lesions > 10 mm, for which hot procedures (polypectomy, EMR, or ESD) will be applied.

Image-enhanced endoscopy with either narrow band imaging or chromoendoscopy with 0.4% indigo carmine spraying will be applied in both arms before polypectomy, to confirm the diagnosis, and after polypectomy, to optically evaluate the complete eradication [23]. The total procedure time, the insertion time, the time to withdrawal, and the time required for polypectomy will be recorded.

### Pathologic examination

All resected specimens will be sent to a central, dedicated pathologist for assessment of complete histologic eradication at all study hospitals. The pathologist will be a gastrointestinal specialist who will be blinded to the clinical information, including randomization. The diagnosis of adenomatous lesions will be based on the 2010 World Health Organization classification of tumors [24].

### Outcome variables

**Fig. 2:**
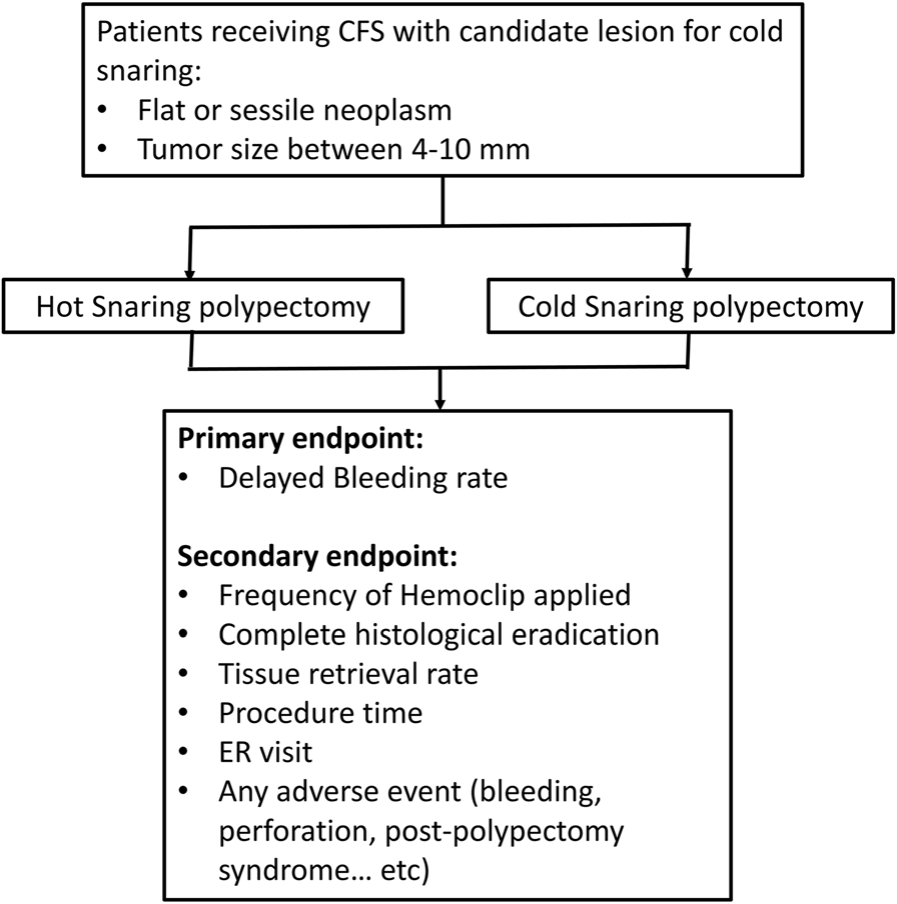
Target lesions are colorectal neoplasms 4–10 mm in size and with flat or sessile morphology. The primary outcome measurement is the risk of delayed bleeding

The primary and secondary outcome measures are shown in Fig. 2. The primary outcome measure is the risk of delayed bleeding within two weeks after polypectomy. Bleeding is defined as any of following:

1. Hemoglobin drops ≥ 2.0 g/dL in comparison with the baseline;

2. Requires blood transfusion;

3. Hematochezia occurs;

4. Requires intervention for hemostasis, including endoscopic hemostasis, transarterial embolization, or surgery.

**Fig. 3:**
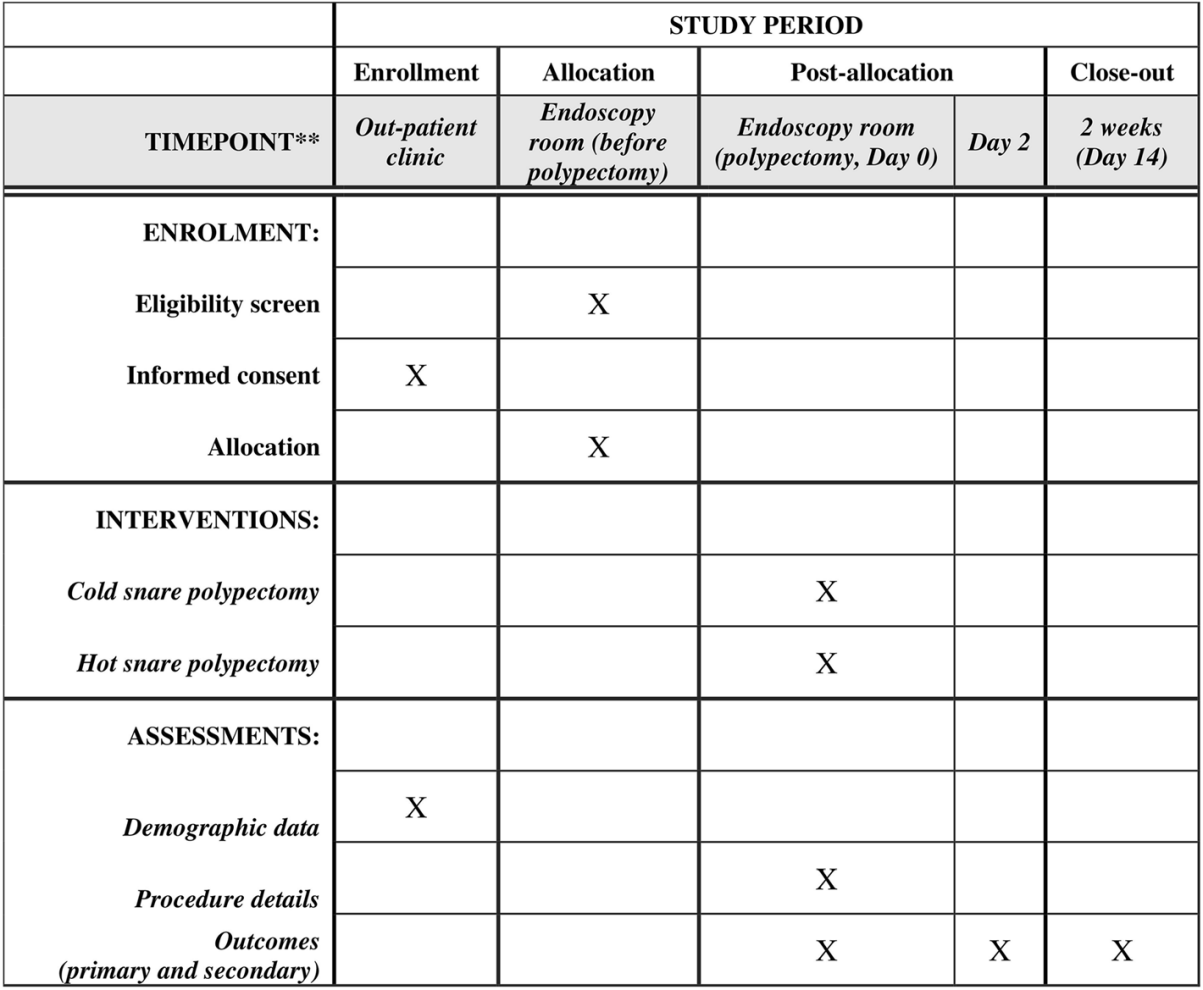
Schedule of enrollment, interventions, and assessments as per Standard Protocol Items: Recommendations for Interventional trials (SPIRIT)

Secondary outcome measures include the number of hemoclip applications, complete histological and visual confirmation of eradication, histopathology of resected polyps, tissue retrieval rate, procedure time, emergency service visits, and other adverse events such as immediate bleeding during the procedure or perforation (Fig. 2). The study nurses/assistants will contact all patients by phone calls on day 2 and day 14 after the colonoscopy procedure to collect these information. Please see Fig. 3 for the scheme of schedule of enrollment, interventions, and assessments.

### Sample size calculation and statistical analysis

This RCT is designed as a two-arm comparison. The risk of delayed bleeding after polypectomy is based on previous publications and is estimated to be 0.6% [25]. The risk of delayed bleeding is assumed to be reduced by 77% in cold snaring polypectomy in comparison with hot snare polypectomy [26]. When the statistical power is set at 80% and the significance level at 0.05, the estimated total sample size is 4258 patients with 2129 in each arm.

The statistical analyses will be performed on an intention-to-treat basis. Values will be expressed as mean ± standard deviation (SD) or median, as appropriate. Comparisons of continuous and categorical data will be made using Student’s *t*-test and the chi-square test, respectively. A *p* value < 0.05 will be considered statistically significant.

## Discussion

Cold snaring polypectomy is expected to reduce the risk of delayed bleeding by avoiding thermal injury. However, previous RCTs failed to prove the protective effect of cold snaring polypectomy because of their limited sample sizes. In this study, we will enroll a much larger sample size compared with previous studies in order to demonstrate the benefit of reducing delayed bleeding by cold snaring polypectomy. In addition, another advantage of cold snaring polypectomy is time savings in comparison with hot snaring polypectomy. This has been explored by previous studies and will be also confirmed in this study.

Complete histologically or optically confirmed eradication is an important outcome to evaluate the efficacy of the therapeutic technique. The reported incomplete resection rate following cold snaring polypectomy is 7–21% [13, 27–29] In contrast, following hot snaring polypectomy the incomplete resection rate is 6.8% for polyps sized 5–9 mm [30]. A recent RCT in Japan included 796 polyps sized in the range of 5–9 mm and concluded that the complete resection rate for cold snare polypectomy is not inferior to that for hot snare polypectomy (98.2% vs 97.4%, respectively) [31]. We will compare the complete histologically and visually confirmed eradications between cold and hot snaring polypectomy. This will help to clarify the relative efficacy of cold snaring polypectomy. Moreover, tissue retrieval rates, the frequency of hemoclip application, and any adverse effects will also be evaluated in the study. Thus, this RCT will provide a comprehensive comparison between cold and hot snaring polypectomy.

Diminutive or small colorectal polyps comprise the majority of lesions that should be removed at colonoscopy. Unfortunately, endoscopists use a variety of techniques to remove diminutive or small lesions and practitioners must identify the most cost-effective and safe techniques to treat such lesions. In this study, we will conduct a comprehensive evaluation of cold snaring polypectomy and the results of this RCT will provide a valuable information for future standardization of cold snaring polypectomy for treating diminutive or small lesions.

## Trial status

The first investigators’ meeting took place on 29 October 2017. The study was registered at ClinicalTrials.gov (NCT03373136) on 14 December 2017. The RCT is in preparation now and will launch in July 2018. Recruitment is expected to end in late 2020.

## Additional file

Additional file 1: SPIRIT 2013 Checklist: Recommended items to address in a clinical trial protocol and related documents. (DOC 120 kb)

## Abbreviations

CRC: Colorectal cancer
EMR: Endoscopic mucosal resection
ESD: Endoscopic submucosal dissection
RCT: Randomized controlled trial
SD: Standard deviation
